# Gene Therapy with p14/tBID Induces Selective and Synergistic Apoptosis in Mutant Ras and Mutant p53 Cancer Cells In Vitro and In Vivo

**DOI:** 10.3390/biomedicines11020258

**Published:** 2023-01-18

**Authors:** Robert L. Fine, Yuehua Mao, Dario Garcia-Carracedo, Gloria H. Su, Wanglong Qiu, Uri Hochfeld, Gwen Nichols, Yong-Liang Li, Richard D. Dinnen, Anthony Raffo, Paul W. Brandt-Rauf

**Affiliations:** 1 Experimental Therapeutics Program, Division of Medical Oncology, The Pancreas Center at Columbia, Herbert Irving Comprehensive Cancer Center, NYPH-Columbia University Medical Center, New York, NY 10032, USA; 2 Department Pathology and Otolaryngology, Herbert Irving Comprehensive Cancer Center, NYPH–Columbia University Medical Center, New York, NY 10032, USA; 3 Department of Environmental Health Sciences, Mailman School of Public Health, Columbia University, New York, NY 10032, USA; 4 School of Public Health, University of Illinois at Chicago, Chicago, IL 60612, USA; 5 School of Biomedical Engineering, Science and Health Systems, Drexel University, Philadelphia, PA 19104, USA

**Keywords:** p14^ARF^ promoter, p14, tBID, apoptosis, gene therapy, p53, Ras

## Abstract

Any gene therapy for cancer will be predicated upon its selectivity against cancer cells and non-toxicity to normal cells. Therefore, safeguards are needed to prevent its activation in normal cells. We designed a minimal p14^ARF^ promoter with upstream Ap1 and E2F enhancer elements and a downstream MDR1 inhibitory element, TATA box, and a transcription initiation site (hereafter p14^ARF^min). The modified p14^ARF^min promoter was linked to bicistronic *P14* and truncated *BID* (*tBID*) genes, which led to synergistic apoptosis via the intrinsic and extrinsic pathways of apoptosis when expressed. The promoter was designed to be preferentially activated by mutant Ras and completely inhibited by wild-type p53 so that only cells with both mutant Ras and mutant p53 would activate the construct. In comparison to most p53 gene therapies, this construct has selective advantages: (1) p53-based gene therapies with a constitutive CMV promoter cannot differentiate between normal cells and cancer cells, and can be toxic to normal cells; (2) our construct does not induce p21^WAF/CIPI^ in contrast to other p53-based gene therapies, which can induce cell cycle arrest leading to increased chemotherapy resistance; (3) the modified construct (p14^ARF^min-p14-tBID) demonstrates bidirectional control of its promoter, which is completely repressed by wild-type p53 and activated only in cells with both *RAS* and *P53* mutations; and (4) a novel combination of genes (p14 and tBID) can synergistically induce potent intrinsic and extrinsic apoptosis in cancer cells.

## 1. Introduction

Pancreatic cancer (PC) is the fourth leading cause of all cancer deaths in the U.S. Approximately 49,500 new cases of PC were diagnosed in 2021 in the U.S. and over 90% of the advanced patients succumb to PC within two years. Only 6% of PC patients, irrespective of initial stage, will survive five years. K-*RAS* mutations (mut K-Ras) have been found in up to 90% of patients with PC, while *P53* mutations have been found in 70–80% of PC patients. Inactivation of the *P16^INK4A^*/*P14^ARF^* gene through hypermethylation, mutation, or deletion occurs in 100% of human PC [1,2,3,4].

These three genetic mutations (K-Ras, p53, p14/p16) could be exploited in such a way that they become an “Achilles Heel”. Thus, a gene therapy utilizing a construct which is activated mainly by mut Ras and repressed by wild-type p53 (wt p53) could be a selective gene therapy for PC, as up to 70–80% of PCs have both mut Ras and mut p53 [2]. This combination of mutations is present in a significant number of cancers, such as non-small cell lung, bladder, and colon. To exploit the combination of *RAS* and *P53* mutations in PC, as well as in other cancers, we constructed a modified p14^ARF^ promoter that is selectively and specifically activated by constitutively activated mut Ras (K-, H- and N-Ras) and inhibited by wt p53. We chose the p14^ARF^ promoter because it has bidirectional control; it is stimulated by mut Ras and repressed by wt p53. Thus, a PC with both mut Ras and mut p53 would preferentially activate this promoter, while normal or malignant cells with either wt Ras or wt p53 would not. The modified p14^ARF^ promoter was bicistronically linked to whole *P14* and *tBID* genes. This synergistically induced both the intrinsic and extrinsic pathways for apoptosis in cancer cells with both mut Ras and mut p53 [5,6,7,8,9].

## 2. Materials and Methods

### 2.1. Cell Culture

**Figure 1:**
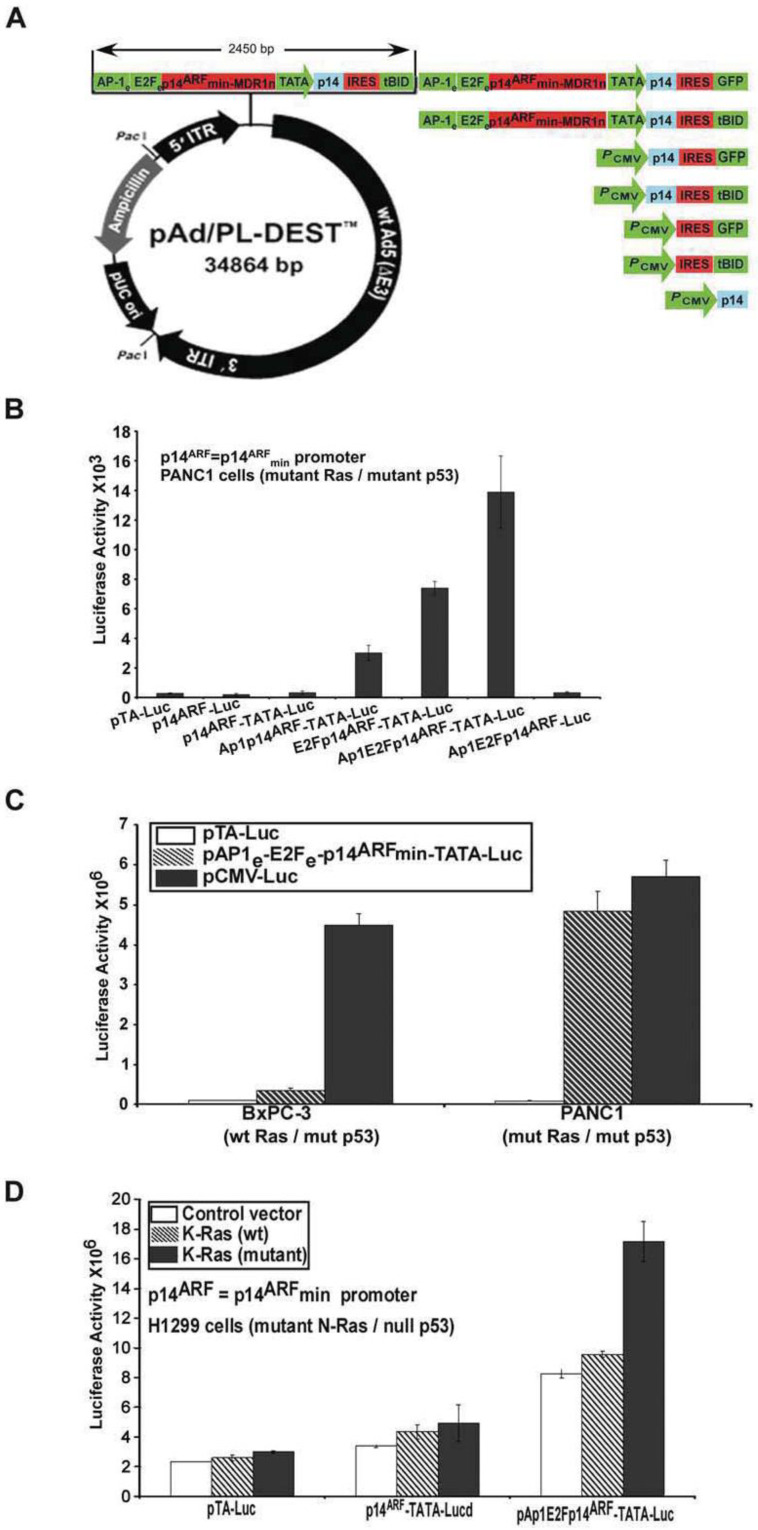
(**A**) Adenovirus vectors diagram. The adenovirus vector pAd/PL-DEST (Invitrogen) was inserted, and fragments were confirmed by sequencing. (**B**) Luciferase assay. PANC-1 cells were transiently transfected with plasmids as indicated for 24 h. Results are presented as the mean relative activity for triplicate transfections. pAP1e-E2Fe-p14^ARF^min-TATA-luc had the highest expression, whereas the same modified minimal p14^ARF^ promoter without a TATA box had very little expression. (**C**) Effects of Ras status upon promoter activation. BxPC-3 cells (wt Ras/mut p53) and PANC-1 cells (mut Ras/mut p53) were transiently transfected with pTA-luc, pAp1e-E2Fe-p14^ARF^min-TATA-luc, and pCMV-Luc for 24 h. The plasmid pAp1e-E2Fe-p14^ARF^min-TATA-luc expression was ≥15 fold higher in PANC-1 than BxPC-3 cells and control. (**D**) Stimulation of the modified p14^ARF^min promoter by mutant and wild type K-Ras. H1299 (null p53, mut N-Ras) lung cancer cells were pre-treated with Ad- pCMV-K-Ras (wt) or Ad-pCMV-K-Ras (mut G12D) for 6 hrs. and then transfected with the above luciferase containing plasmids for another 20 hrs. The plasmid pAP1e-E2Fe-p14^ARF^min-TATA-luc expression was 11-fold higher (220%) in Ad-mut-K-Ras G12D as compared to Ad-wt-K-Ras cells (20%). The controls pTA-luc and p14^ARF^min-TATA-luc without enhancer elements (Ap1e and E2Fe) showed minimal luciferase activity.

All human cell lines and their in vitro requirements were obtained from ATCC and tested in an exponential growth phase (Manassas, VA, USA) (Table S1A,B). Construction of pCMV-p14, pCMV-tBID, pCMV-p14-tBID, and pCMV-p14-GFP vectors cDNA for the human *p14* and tBID were generated by RT-PCR with primer sets derived from sequences found in the NCI Nucleotide Database. The GFP gene was generated by PCR using pEGFP-N2 (BD Biosciences Clontech, Palo Alto, CA, USA) as a template. The fragments were inserted into the corresponding multiple cloning sites A or B, or both cloning sites of the vector pIRES (BD Biosciences Clontech, Palo Alto, CA, USA). This was utilized to make corresponding vectors pCMV-p14, pCMV-tBID, pCMV-p14-tBID, and pCMV-p14-GFP, as shown in Figure 1A.

### 2.2. Construction of the Modified p14^ARF^min Promoter Expression Vectors

The human p14^ARF^ gene promoter region was originally identified as a 5.6-kb CpG-rich region 5′ upstream of the p14 first exon (1β) [10]. However, investigators have used the first 800 bases immediately upstream of the transcription initiation site, and have shown that this region is sufficient to confer AP1 and E2F regulation in a sensitive reporter system [9]. This region, which we termed the minimal human p14^ARF^ promoter (p14^ARF^min), was the starting promoter region in our p14^ARF^ promoter constructs. The modified p14^ARF^min promoter was amplified by PCR from human genomic DNA using the published sequence [9]. Forward primer, 5′- CCATTAATGTCGACAGCTCCGGCAGCGC-3′ and reverse primer, 5′-ACTCGAGATCTCCGCCCCG.

CAGGCGCGCA-3′ were used along with the restriction sites *AseI* and *XhoI* to replace the CMV promoter of pIRES vector with the p14^ARF^ promoter.

Since attempts at expressing GFP in the mut Ras/mut p53 pancreatic cell lines were unsuccessful with the p14^ARF^ promoter (data not shown), a promoter analysis program (Cister: Cis-element Cluster finder, Boston University) was used to detect the presence and efficacy of various transcription factor binding elements and promoter features. We did not use the whole p14^ARF^ promoter because it was too large (5.6 kb) for use in an adenovirus vector, which holds a maximum of 5 kb. While this program identified an E2F potential binding site [9], its homology to the consensus E2F site was low. Additionally, an AP-1 site, the “TATA” box, and the transcription initiation site, while present, also showed poor homology with consensus sites. Two reporter plasmids, pAP1-Luc and pE2F-Luc (BD Bioscience Clontech, Palo Alto, CA, USA), were then used to transfer each of the transcription factor binding elements, E2F and Ap1 enhancers, to a 5′ region upstream of the p14^ARF^ promoter. The E2F enhancer site, containing 4 tandem repeat E2F elements totaling 70 bp, and the AP1 enhancer site, containing 4 tandem repeat Ap1 elements totaling 50 bp, were amplified by PCR and inserted upstream of the p14^ARF^ promoter. Sequencing was performed to confirm the direction of orientation and the integrity of the promoter’s structure. Additionally, using designed primers and serial PCR amplifications, the sequence of the downstream p14^ARF^ was modified by inserting the TATA box and transcription initiation sequence, which shares more homology with the consensus TATA box sequence than that found in the p14^ARF^ promoter. The TATA box was important in transcription because the TATA factor binding to the region recruits additional factors to initiate transcription. The constructed promoter Ap1e-E2Fe-p14^ARF^-TATA–transcription initiation site was called the modified p14^ARF^min promoter, or simply called the p14^ARF^. The modified promoter was inserted into the replaced CMV promoter site to make the respective plasmids p14^ARF^-p14 or tBID or p14-IRES-tBID and p14-IRES-GFP (Figure 1A–D).

### 2.3. Ap1e-E2Fe-p14^ARF^min-MDR1n-p14-tBID Construction

Based on the above construct and PCR method, we amplified the MDR1n negative regulatory element (50 bp) core sequence TTGGCTGGGCAGGAACAGCGCCGGGGCGTGGGCTGAGCACAGCGC [11] with compatible ends. The MDR1n 5′ site is compatible with the 3′ of p14^ARF^ promoter and the MDR1n 3′ is compatible with the 5′ of the TATA box. Then, using PCR, we joined together the construct Ap1e-E2Fe-p14^ARF^min-MDR1n-TATA-p14-IRES-tBID; for simplicity, the entire construct is called p14^ARF^-MDR1n-p14-tBID. The bp size of the construct was intentionally restricted to minimize the size of the construct so as to be insertable into the Ad5 viral vector. The entire construct (Ap1e-E2Fe-p14^ARF^min-MDR1n-TATA-p14-IRES-tBID) consists of 2450 bp, less than half of the size of the full p14^ARF^ promoter (Figure 1A).

The Virapower Adenoviral Promoterless Gateway expression kit (Invitrogen, Corp., Calsbad, CA, USA) was used to assemble replication-defective adenoviral particles containing the above promoter/product construct. Viral construction plasmids, generation of particles by helper cells, measurement of focus forming units, and multiplicity of infection (MOI) units were accomplished following the manufacturer’s instructions. Cell lines were first tested with various MOIs for their ability to express GFP (pCMV-p14-GFP) in mut Ras and mut p53 PC lines within a 48-h period. Once saturation conditions were observed (data not shown), other constructs containing tBID were constructed and tested, and these are shown in the figures. Characteristics of each cell line and test results in 21 cell lines and human CFU-GEMM colonies with different Ras and p53 status are summarized in Supplementary Table S1A,B.

### 2.4. Luciferase Assay

Cell lysates were assayed for luciferase activity and quantitated by a 20/20^n^ Luminometer System (Promega, Madison, MI, USA).

### 2.5. Flow Cytometric Analysis for Cell Cycle and Apoptosis

All 21 cell lines were treated with the indicated Adeno-viral construct for either 24 or 48 h, and the cells were then stained by propidium iodide (Sigma, St. Louis, MO, USA) following the instruction and analyzed with a FACSCalibur flow cytometer (BD Biosciences, Palo Alto, CA, USA) to quantitate subdiploid DNA.

### 2.6. Western Blot Analyses

Western blots were carried out using RIPA-containing whole-cell extracts from various cell lines, as stated. Antibodies to PARP (sc-8007), Bcl-2 (sc-509), BAK (sc-832), Bax (sc-493), p14 (sc-8613), Fas (sc-65316), BID (sc-6538), and Fas-L (sc-6237) were from Santa Cruz Biotechnology, Dallas, TX, USA (except antibody to tBID (44-443, Biosource Labs, Highland, UT, USA) and alpha tubulin (T-5168, Sigma-Aldrich, St. Louis, MO, USA)).

### 2.7. Human CFU-GEMM Stem Cell Assay

Bone marrow stem cells were obtained from normal volunteers who gave written IRB-approved consent for bone marrow stem cell (CD34+) collection. The cultures in M3434 medium containing agar were prepared in 24-well plates incubated at 37 °C in a 5% CO_2_ incubator. The numbers of burst-forming units of erythroid (BFU-E), CFU-GM, and colony-forming units of CFU-GEMM progenitor-derived colonies were determined at days 12–14 of culture. Cell colonies that were counted as positive had ≥50 cells/colony, and the reader was blinded to the groups. We had previously found that adenovirus receptors were present in marrow stem cells utilizing Ad-pCMV-GFP vectors and demonstrated ample internalization and synthesis of GFP (data unpublished).

### 2.8. Animal Studies

#### 2.8.1. Human Lung Cancer Xenograft Model

Effects of Ad-p14^ARF^-MDR1n-p14-tBID on tumor growth kinetics were tested in two human lung cancer cell lines in athymic female mice. Tumor volumes were measured over two weeks after the subcutaneous (SC) implantation of an osmotic Alzet pump, which delivered 0.2 mL virion solution (10^9^/mL virions) over a 14-day period. 1 × 10^6^ cells were injected SC into the hind flank of mice with either the lung H460 (mut K-Ras/wt p53) or lung H1299 (mut N-Ras/null p53) cell lines. When the tumors reached 100 mm^3^ in the first experiment, the Alzet pumps were implanted juxtaposed to face the tumors, but not in physical contact. Tumors were measured 3 times a week over a 2-week period. For the second experiment, the same tumors were allowed to grow to a larger size (200 mm^3^), and then the animals received intraperitoneal (IP) injections every 4 days over a 25-day period (7 injections). This experiment was designed to test the efficacy of Ad-p14^ARF^-p14-BID therapy when the tumor was larger and with anterior abdominal IP injection distal to the dorsal hind limb tumor site.

All experiments were performed twice, and in each experiment, *n* = 14 for the Ad-p14^ARF^-MDR1n-p14-tBID group and *n* = 10 for the GFP group. A Student’s *t*-test for differences in tumor volumes showed a *p* value ≤ 0.005 for the first experiment with H1299 (mut Ras/null p53) vs. the H460 (mut Ras/wt p53) mice receiving Ad-p14^ARF^-MDR1n-p14-tBID. In the second experiment with IP injections distal to larger tumors, the H2199 vs. H460 groups receiving p14-tBID and control Ad-GFP, respectively, had a *p* value < 0.01.

#### 2.8.2. Pancreatic Cancer in the Mut K-Ras/Mut p53 KPC GEMM Mouse Strains and Endpoint Treatment Procedure

We interbred the conditional *LSL-Kras^G12D/+^*; *LSL-Trp53R^172H/+^*; *Pdx-1-Cre* mut animals (KPC) as previously reported [12,13,14,15]. Treatments began at 4 months of age, which corresponds to a month before the median survival of these mice [13], and ended at 6 months of age, when all animals have gross PC. The mice were treated via IP injection with 200 µL (10^9^/mL virions) of Ad-p14^ARF^-MDR1n-p14-tBID (*n* = 11) or Ad-GFP (*n* = 10; control) 3 times a week for 2 months. All experiments were conducted in compliance with protocols approved by the Institutional Animal Care and Use Committee of Columbia University Medical Center (CUMC). The mice were a mixed 129/SvJae/C57Bl/6 background, and all the experiments were carried out in littermates. Mice were weighed prior to sacrifice and the pancreas was weighed at necropsy.

#### 2.8.3. Measurement of Tumor Foci

Tissues were harvested and fixed in 10% formalin overnight and embedded in paraffin. Then, 5-µm slides were cut and routine H&E staining was performed by the Histology Service Center at the Core Facilities of the Herbert Irving Comprehensive Cancer Center at Columbia. To determine the size of each tumor focus, the diameter of the largest tumor focus in three serial H&E-stained sections (50 µm from each other) was measured using a 10x objective with a field width of 1 mm. The average value was calculated for each group. We also determined the sum of the maximum linear dimension of tumor foci in three serial H&E-stained sections (50 µm from each other) using a 10× objective with a field width of 1 mm. The average value was calculated for each group. The Student’s *t*-test was used to calculate the *p* values.

#### 2.8.4. Imaging of KPC Pancreatic Tumors by High-Resolution Ultrasound

High resolution ultrasound (US) imaging of normal and tumor mouse pancreas using the VisualSonics Vevo2100 High Resolution System with a VisualSonics MS-550D ultrasound transducer (35 MHz RMV)(VisualSonics, Inc., Toronto, ON, Canada) was performed as described [16,17]. Then, 3D images were produced using VisualSonics Vevo2100 3D motor arm to collect serial images at 0.25 mm intervals through the entire thickness of the tumor. Tumors were outlined on each 2D image and reconstructed to quantify the 3D volume using the integrated Vevo2100 software package.

#### 2.8.5. Survival Study

Enrollment of KPC mice was based on tumor size (measured by ultrasound). Specifically, ultrasound imaging was performed initially on animals 8–12 weeks old for detection of tumor and/or cystic lesion of the pancreas. Once the tumors were visualized, they were scanned in an axial orientation and long and short axes of the tumor were averaged to give a mean diameter. Mice with mean tumor diameters of 2–5 mm (average: 3 mm) were enrolled. To measure the tumor growth before and after enrollment, high-resolution ultrasound was performed once a week. Enrolled mice were treated by IP injection with 200 µL of the concentrated solution of Ad-p14ARFmin-MDR1n-p14-IRES-tBID (*n* = 10) or Ad-GFP (*n* = 11; as the control) 3 times a week until endpoint criteria were met. Endpoint criteria included the development of abdominal ascites, severe cachexia, significant weight loss exceeding 20% of initial weight, or extreme weakness or inactivity. Survival curves were calculated using the Kaplan–Meier product–limit estimate. Differences between survival times were analyzed by the log-rank method.

## 3. Results

### 3.1. A Modified p14^ARF^ Promoter Is Specifically Expressed in Mutant Ras and Mutant p53 Cancer Cell Lines

To assess the activation of the p14^ARF^ promoter (5.6 kb) in the PANC-1 cancer line, which carries both mut K-Ras/mut p53, we constructed various modified p14^ARF^ promoters, as shown in Figure 1A,B, based on the promoter’s linkage [9] with a luciferase reporter gene construct. We found that a modified minimal p14^ARF^ promoter (p14^ARF^min-850 bp) linked upstream to Ap1 (45 bp) and E2F (70 bp) enhancers (both activated more by mut than wt Ras) at its 5′ end and the transcription initiator and binding site TATA box (20 bp) downstream at the 3′ site induced the highest luciferase activity (p14^ARF^min promoter). If the TATA transcription initiator and binding site were removed, mut K-Ras could not induce activation of transcription, though it still contained both Ap1 and E2F enhancer elements (Figure 1B). The minimal p14^ARF^ promoter alone, without an added transcription initiator and TATA box, could not initiate transcription in PANC-1 cells. In the full-length p14^ARF^ promoter, its atypical transcriptional initiation and binding site interact with the p14^ARF^ promoter upstream transcription responsive elements to initiate transcription. p14^ARF^ promoters with TATA boxes but without the enhancers AP1 and E2F also displayed low expression of luciferase from mut K-Ras (Figure 1C). To test the differential activation abilities of wt K-Ras and mut K-Ras (G12D) on the modified p14^ARF^ promoter (Ap1e-E2Fe-p14^ARF^-TATA-luc), we constructed wt K-Ras plasmids from cDNA of CCD-27sk cells (normal human skin fibroblasts) and mut K-Ras plasmids from cDNA of PANC-1 cells. These were transfected into H1229 cells by the Adenovirus 5 (Ad) vector. As shown in Figure 1C, mut K-Ras was 2.0-fold more efficient for activating the modified p14^ARF^ promoter than wt K-Ras in the luciferase assay at equal multiplicity of infection (MOI). WT K-Ras increased luciferase activity by 15–20% over control Ad-vector, while mut K-Ras increased activity over 220% above control. To further prove the necessity of mut K-Ras in activating the modified p14^ARF^ promoter, we transiently transfected Ap1e-E2Fe-p14^ARF^-TATA-Luc (p14^ARF^min-Luc) constructs and control plasmids into the human pancreatic cancer line BxPC-3 cells (wt K-Ras/mut p53) and PANC-1 cells (mut Ras/mut p53). As shown in Figure 1D, p14^ARF^min-Luc was expressed only in PANC-1 cells but not in BxPC-3 cells, as the BxPC-3 cells have wt K-Ras.

### 3.2. Bidirectional Control of the Modified p14^ARF^min Promoter

**Figure 2:**
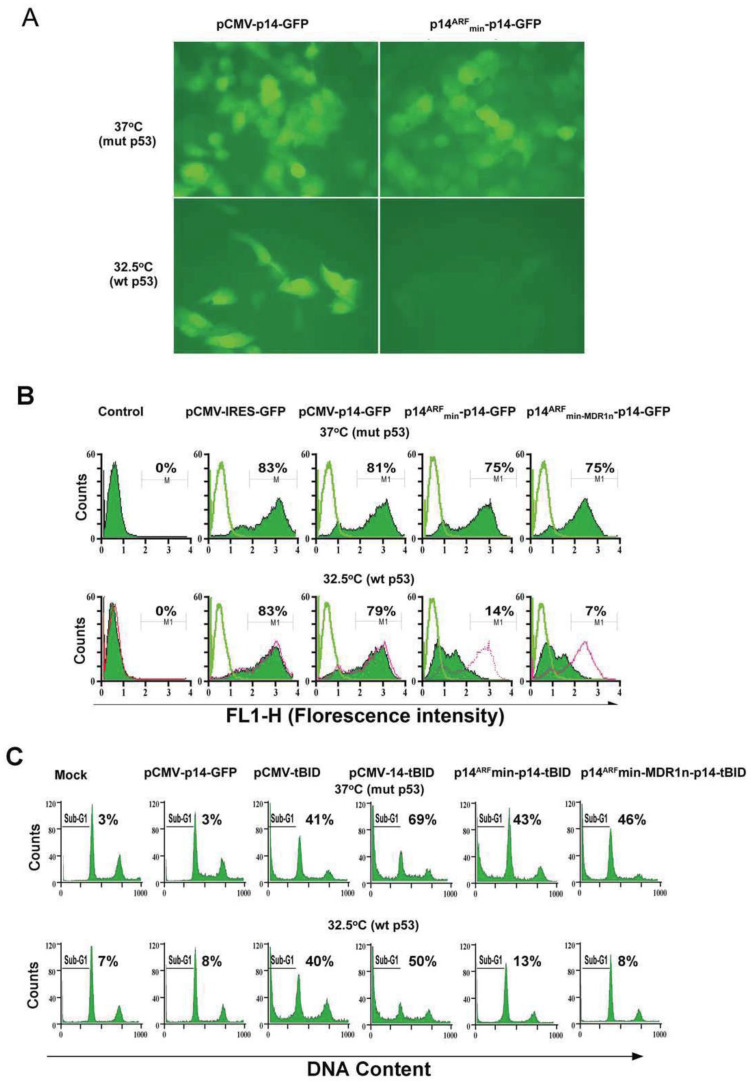
(**A**) GFP expression in H1299 lung cancer-p53^TS^ stable cell line. H1299/p53-143#6 cells were infected with 5 MOI of adenoviral vectors as indicated. Cells were fixed with 4% formaldehyde 24 h after infection and were analyzed with fluorescence microscopy. GFP expression induced by the Ad-p14^ARF^min-p14-GFP was nearly totally inhibited in the engineered lung cancer H1299 cells at wt p53 temperature (32.5 °C). (**B**) Quantitation of GFP expression levels in H1299 lung cancer-p53^TS^ stable cell line. H1299/p53-143#6 cells were infected with adenoviral vectors (5 MOI) as indicated. At 48 h post-infection, the H1299 ts p53 cells infected with Ad-p14^ARF^-p14-GFP had significantly decreased expression at wt p53 (32.5 °C) as compared to mut p53 37 °C (14% vs. 75%), respectively. Addition of the MDR1n to the above construct further reduced GFP expression from 75% (37 °C) to 7% (32 °C). (**C**) Induction of apoptosis by modified p14^ARF^min-p14-tBID is inhibited by wild-type p53 (p14-tBID = p14-IRES-tBID). H1299/p53^TS^ (V143A) cells were infected with 5 MOI of virus containing vectors as indicated. Cells were harvested at 48 h. after infection and analyzed by flow for apoptosis by the PI assay (subG1 DNA). One of three independent experiments is shown, and the percentage of apoptotic cells is indicated. The addition of the MDR1n to the construct further suppressed apoptosis to baseline control levels (8%). Additionally, at 37 °C (mut p53), there was synergistic cytotoxicity when p14-IRES-tBID was concomitantly expressed, but no synergism occurred at the 32 °C (wt p53) where suppression of the construct occurred by wt p53.

To assess whether the modified promoter could be inhibited by wt p53, we constructed an adenovirus containing the modified p14^ARF^ promoter linked to the p14 gene and GFP with an internal ribosome entry site (p14-IRES-GFP). This bicistronic expression construct is called Ad-modified-p14^ARF^min-p14-IRES-GFP (abbreviated now as Ad-p14^ARF^min-p14-GFP). Within this construct, the *P14* gene acts as a fail-safe to ensure that if the modified p14^ARF^ promoter is activated in non-malignant cells containing activated wt Ras and low levels of wt p53, the increased p14 protein will inhibit HDM-2-mediated ubiquination and proteosomic degradation of wt p53, leading to increased levels of wt p53. This increase of wt p53 produces feedback inhibition of the p14^ARF^ promoter. GFP was utilized as a reporter gene to monitor live cell expression. H1299 cells carrying endogenous mut N-Ras and null p53 were first stably transfected with temperature-sensitive p53 (p53^TS^; V143A) and further transduced with Ad-p14^ARF^min-p14-GFP. Substantial expression of GFP was observed at mut p53 permissive temperature (37 °C), but not at wt p53 permissive temperature (32.5 °C) (Figure 2A). Quantitative analysis of the GFP expression showed repression of GFP fluorescence levels from 75% to 14% when switched from 37 °C (mut p53) to 32.5 °C (wt p53) (Figure 2B). The control construct with a CMV promoter shows high expression at both temperatures (5 MOI).

Because the construct was not fully repressed at 32 °C (wt p53) (14% expression of GFP), the 50 bp MDR1 negative regulatory element (MDR1n) was inserted downstream of the p14^ARF^ promoter and upstream of the TATA box (Figure 2B, last right panel). The MDR1n element is potently inhibited by wt p53 and not by mut or null p53. This vector with MDR1n, now AP1e-E2Fe-p14^ARF^min-MDR1n-TATA-p14-IRES-GFP (p14^ARF^min-MDR1n-p14-GFP), showed further reduction in expression from 14% to 7% at 32°C (wt p53) (Figure 2B). The various constructs that were tested above and the final vectors are shown in Figure 1A,B.

### 3.3. Bicistronic Gene Products Which Induce Synergistic Apoptosis

In order to construct gene products which: (1) induce synergistic apoptosis selectively in cancer cells with both mut Ras and p53; (2) activate both intrinsic and extrinsic pathway of apoptosis; and (3) retain the fail-safe mechanism utilizing the *P14* gene to prevent activation in cells with low levels of wt p53, we evaluated the expression of a variety of pro-apoptotic genes with whole p14 including Bax, Bak, Fas, BID and truncated BID (tBID). Among all the pro-apoptotic genes, tBID expressed concomitantly with p14 produced the highest levels of synergistic cytotoxicity (data shown later). One of the major advantages of the modified p14^ARF^ promoter (Ap1e-E2Fe-p14^ARF^min-MDR1n-TATA) is its unique ability to be bidirectionally controlled. Constitutively activated mut Ras stimulates the AP-1 and E2F enhancers as well as the p14^ARF^ promoter. However, wt p53 will potently repress the p14^ARF^ promoter and MDR1n elements of the promoter (Figure 1A).

To demonstrate the synergy of p14-tBID and to further demonstrate that Ad-p14^ARF^min-p14-tBID ± MDR1n can be activated by mut Ras and repressed by wt p53 in situ, we infected the stably transfected H1299 p53^TS^ (V143A) lung cancer line with Ad-pCMV-p14-IRES-GFP, Ad-pCMV-IRES-tBID, Ad-pCMV-p14-IRES-tBID and Ad-p14^ARF^min-p14-IRES-tBID ± MDR1n. As shown in Figure 2C, Ad-pCMV-p14-tBID induced significant levels of apoptosis at both temperatures: 69% at 37 °C (mut p53) and 50% at 32 °C (wt p53) at MOI of 5. The Ad-p14^ARF^min-p14-tBID induced apoptosis in 43% of cells at 37 °C but killed 13% of cells at 32 °C. Insertion of MDR1n into the construct induced apoptosis in 46% of the cells at 37 °C (mut p53) and reduced cell death at 32 °C to control basal levels (8%), indicating complete suppression of mut N-Ras stimulation by wt p53 (32 °C). These results were also replicated in the prostate cancer PC-3 null p53 and PC-3 p53^TS^ (V143A) cell lines (data not shown).

Only the bicistronic expression of both tBID and p14^ARF^, driven either by a pCMV or modified p14^ARF^min promoter, induced synergistic cell death. However, this did not occur with p14 or tBID alone in the above H1299 ts p53 lung cancer line (Figure 2C) and PC-3 prostate cells.

### 3.4. Modified p14^ARF^-MDR1n-p14^ARF^-tBID Expression Selectively Killed Cancer Cells with Both p53 and Ras Mutations

To determine if the MDR1n regulatory element could further decrease the Ad-p14^ARF^-p14-tBID construct’s activation in human cancer cell lines with a different Ras and p53 status, we constructed the modified p14^ARF^-p14-tBID construct ± MDR1n. We infected 24 malignant and non-malignant cell lines, each with a different Ras and p53 status, with Ad-pCMV-p14-IRES-GFP, Ad-pCMV-IRES-tBID, Ad-pCMV-p14-IRES-tBID, Ad-p14^ARF^min-p14-IRES-tBID, and Ad-p14^ARF^min-MDR1n-p14-IRES-tBID (hereafter: p14^ARF^min-MDR1n-p14-tBID; with the MDR1n element or p14^ARF^min-p14-tBID; without MDR1n element).

**Figure 3:**
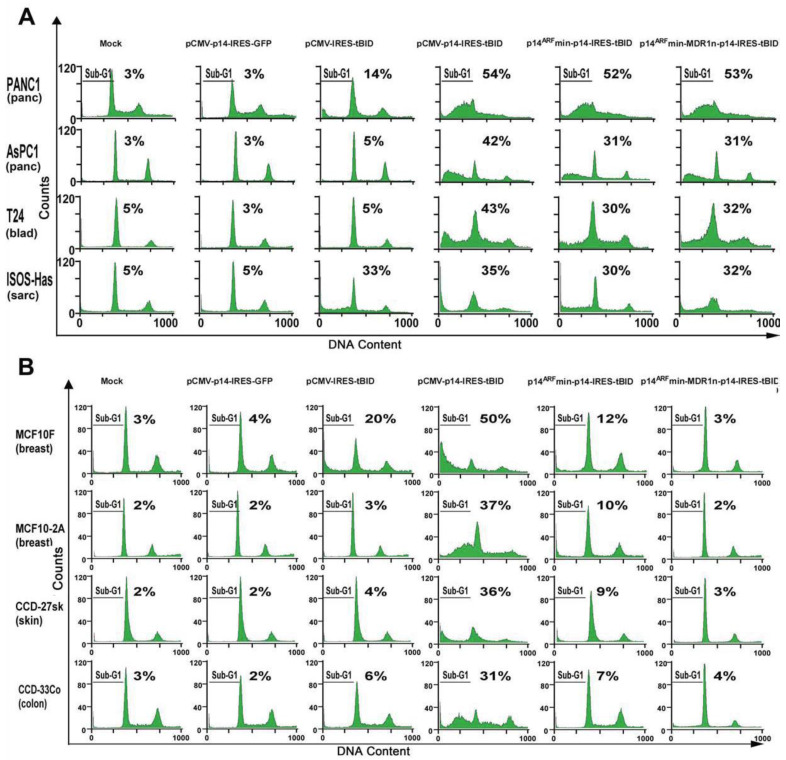
Induction of apoptosis by modified p14^ARF^min-p14-tBID is dependent upon mut Ras/mut p53 status. (**A**) Malignant cell lines with mut Ras/mut p53. PANC-1, AsPC1 (both pancreatic), T24 (bladder) and ISOS-Has (angiosarcoma) are all malignant cell lines with endogenous mut Ras and mut p53, confirmed by cDNA sequencing in our lab. Cells were harvested 48 h after infection and analyzed by flow for apoptosis by PI analysis. The construct pCMV-p14-tBID induced apoptosis in the range of 35% to 54%, while the modified p14^ARF^min-p14-tBID and p14^ARF^-MDR1-p14-tBID induced apoptosis in the range of 31% to 53%. (**B**) Modified p14^ARF^-p14- tBID does not induce apoptosis in non-malignant cell lines. MCF10F, MCF10F-2A (both breast), CCD-27SK (skin), and CCD-33Co (colon) are all nonmalignant lines with wt Ras/wt p53. Cells were harvested 48 h after infection and subjected to PI analysis. The construct pCMV-p14-tBID induced apoptosis in the range of 31% to 50%; the modified p14^ARF^min-p14-tBID induced apoptosis with range of 7% to 12%, and with MDR1n reduced apoptosis to 2% to 4% (control levels).

The human cancer cell lines with mut Ras/mut p53 included: PANC-1, AsPC-1 (both pancreatic), T24 (bladder), and ISO-Has (angiosarcoma) (Figure 3A), and the above mutations were confirmed by DNA sequencing performed in our lab of the entire Ras and p53 exons. After adenovirus exposure to the above cell lines for 48 h, Ad-p14^ARF^min-p14-tBID and Ad-p14^ARF^min-MDR1n-p14-tBID induced apoptosis ranging from 30% to 52%, similar to the Ad-pCMV-p14-tBID construct which induced 35% to 54% apoptosis (Figure 3A). The addition of the MDR1n negative regulatory subunit did not appreciably alter the cell death produced by any of the above constructs, as expected, because these lines have mut p53. Synergistic cytotoxicity was noted when p14 and tBID were concomitantly expressed as compared to expression of either gene alone in all four cell lines. The combined expression led to a three- to five-fold increase in cytotoxicity over p14 or tBID alone (i.e., PANC-1: cytotoxicities-p14-3%, tBID-14%, p14-tBID-54%). This synergistic cytotoxicity occurred with either the pCMV or modified p14^ARF^min promoter-driven constructs (Figure 3A).

To assess whether the Ad-p14^ARF^min-p14-tBID ± MDR1n can be activated in malignant cells with mut Ras/wt p53, we infected various cell lines with the same pCMV or modified p14^ARF^min promoter-driven constructs, as above. In the HCT116 colon cancer and the H460 lung adenocarcinoma cell lines, the modified p14^ARF^ construct without the MDR1n produced 20% and 3% cell death, respectively (Supplementary Figure S1A). The modified p14^ARF^ construct with MDR1n produced 10% and 2% cell death in H460 and HCT116 cell lines, respectively. These two cell lines have mut Ras/wt p53. Ad-pCMV-p14-tBID as a control produced 38% and 56% killing in the HCT116 colon and H460 lung lines, respectively (Supplementary Figure S1A). These results suggest that the addition of MDR1n to the construct allowed low levels of wt p53 to further inhibit the activation of the construct by mut Ras to near baseline control levels. The concomitant expression of CMV-driven p14 and tBID in these cell lines again led to synergistic cytotoxicity as compared to the singular expression of either gene. However, this synergy was only present in the CMV-driven construct because wt p53 inhibited the p14^ARF^ promoter and MDR1n element expression of p14 and tBID.

In malignant cell lines BxPC-3 (pancreatic, wt Ras/mut p53), MB231 (breast, heterozygous mut/wt Ras and mut p53), and A431 (squamous cell carcinoma, wt Ras/mut p53), none of the constructs (Ad-p14^ARF^min-p14-tBID ± MDR1n) induced significant apoptosis (2% to 4%) (Supplementary Figure S1B). However, the control Ad-pCMV-p14-tBID induced apoptosis in 49% to 85% with the same MOI. These results suggest that the modified p14^ARF^min promoter, regardless of the inclusion of MDR1n, is not activated by wt Ras or heterozygous mut Ras (MB231). The synergistic cytotoxicity from concomitant p14-tBID expression was three- to six-fold higher than expression of singular p14 or tBID alone (Supplementary Figure S1B).

In the malignant cell lines with both wt p53 and wt Ras (MSTO-211H mesothelioma, MCF-7 breast, and HEAND liver angiosarcoma), the construct without MDR1n produced 3%, 10%, and 6% cell death, respectively (Supplementary Figure S1C). The construct with MDR1n further reduced cytotoxicity to baseline levels. In the MSTO-211H, MCF-7, and HEAND cell lines, the individual expression of p14 and tBID with a CMV promoter had 1%, 4%, and 4% cytotoxicity for p14 and 18%, 20%, and 20% cytotoxicity for tBID, respectively. Whereas the concomitant expression of CMV-driven p14-tBID led to synergistic cytotoxicity (41%, 50%, and 55%), this did not occur in the modified p14^ARF^min promoter-driven constructs, in which activation was not possible due to the wt Ras/wt p53 status of the cell lines. All the malignant cell lines tested are summarized in Supplementary Table S1A.

**Figure 4:**
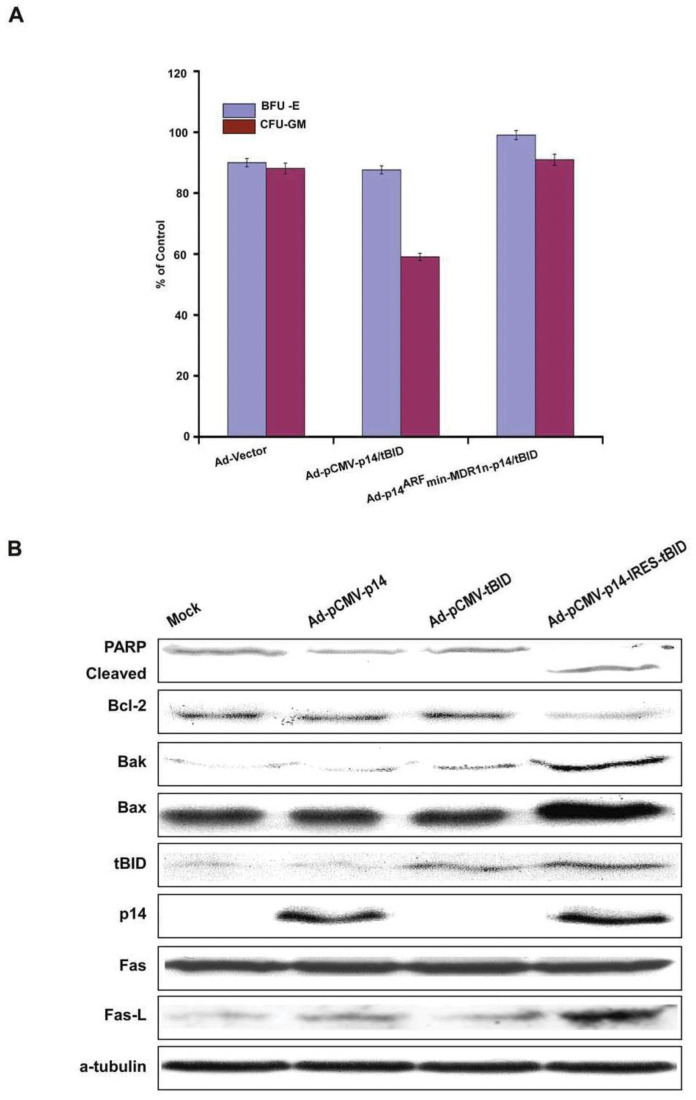
(**A**) CFU-GEMM studies. The human bone marrow peripheral stem cell assay (granulocytes/erythroid/monocyte/macrophage CFU-GEMM) tested the cytotoxicity of various adenoviral constructs. The exposure time to viral vector was for the entire experiment, up to 10–14 days. The pCMV-p14-tBID produced 10% toxicity to BFU-E colonies and 42% toxicity to CFU-GMM colonies. The p14^ARF^-MDR1-p14-tBID produced 5% cell death to BFU-E colonies and 3% toxicity to CFU-GMM colonies (baseline). Ad-5 vectors alone had 10% cytotoxicity. Standard error of mean (S.E.M) bars are on each column from three experiments each done in triplicate. (**B**) Western analysis of apoptotic proteins. Western analysis was done 48-h after PANC-1 cells were infected with each vector indicated. Only p14-tBID together induced PARP cleavage, decreased Bcl-2, and increased Bak, Bax, and Fas-L. However, Fas did not change. The results shown are from the pCMV-driven constructs, and the Ad-p14^ARF^-MDR1n-p14-tBID construct showed identical results in the PANC-1 cell line (mut Ras/mut p53).

Thus, the MDR1n element, along with the modified p14^ARF^min construct, had no inhibitory effect in cell lines with mut Ras/mut p53 and had a nearly complete inhibitory effect in the cancer lines with mut Ras/wt p53 and wt Ras/wt p53 cancer cell lines. However, its purpose in this construct was to provide an additional fail-safe against activation of the construct in normal or non-malignant cells. To test whether modified p14^ARF^min promoter can be activated in non-malignant rapidly dividing cells with wt p53 and activated wt Ras, we infected the construct into non-malignant MCF-10F and MCF10-2A (both breast) and CCD-27sk (skin fibroblast) and CCD- 33Co (colon) cell lines. As shown in Figure 3B, Ad-p14^ARF^min-p14-tBID induced apoptosis ranging from 7% to 12% in non-malignant cells, whereas the same construct with MDR1n further reduced apoptosis to baseline ranging from two to four percent. In comparison, equal MOIs of Ad-pCMV-p14-tBID induced cell death in 31% to 50% of cells in the same non-malignant lines with wt Ras/wt p53 (Figure 3B and Supplementary Table S1B). In these non-malignant cell lines with wt Ras/wt p53, synergistic cytotoxicity was detected only with CMV-driven concomitant expression of p14 and tBID as compared to each gene expressed alone. This synergy ranged from three- to seven-fold and was not evident in the cells exposed to the modified p14^ARF^min promoter-driven construct. To test normal, rapidly proliferating cells, we infected the modified p14^ARF^min and CMV promoter-driven constructs in human marrow CD34+ stem cells for erythroid (BFU-E) and granulocytes/monocytes/macrophage colonies (CFU-GEMM) from normal volunteers who signed consent forms approved by Columbia’s IRB. These rapidly proliferating normal marrow stem cells contain activated wt Ras and wt p53. The p14^ARF^min-p14-tBID construct induced only three percenttoxicity in BFU-E and six percent toxicity in CFU-GMM stem cell colonies ± MDR1n. The CMV-driven construct induced 12% and 42% toxicity in BFU-E and CFU-GMM colonies, respectively (Figure 4 and Supplementary Table S1B).

These results were further supported by our findings on infection of five different constructs into PANC-1 cells, i.e., (mutRas/mutp53), Ad-vector, Ad-pCMV-p14, Ad-pCMV-tBID, and Ad-pCMV-p14-tBID for 48 h.

Of these constructs, only the bicistronic expression of p14 and tBID induced PARP cleavage, significantly increased levels of Fas ligand (Fas-L), Bak and Bax, and decreased levels of Bcl-2 (Figure 4B). p14 or tBID alone did not decrease Bcl-2 levels or increase Bax, Fas, or PARP cleavage, but did induce a modest increase of Bak and Fas-L expression. Similar protein changes were induced in the ASPC-1 pancreatic cell lines after infection with Ad-pCMV-p14-tBID (data not shown; they are available on request to the corresponding authors). These data suggested that the mechanism by which the construct p14-tBID synergizes involved the intrinsic (Bak, Bax, Bcl-2) and extrinsic (Fas-L, PARP cleavage and Caspase-8 activation) pathway of apoptosis. Levels of p21^waf/cip-1^ did not change from expression of p14, tBID, or concomitant p14-tBID, as expected (data not shown).

To further explore the extrinsic pathway for apoptosis, H1229 lung cells (mut N-Ras/mut p53) were infected with Ad-pCMV-p14-tBID. This increased caspase-8 activity 6.9-fold, while the Ad-pCMV-tBID construct increased caspase-8 activity by 4.2-fold and the Ad-pCMV-p14 construct had no effect upon caspase-8 activity levels (Supplementary Figure S2A). The modified p14^ARF^min-p14-tBID ± MDR1n produced a 6.7-fold and 6.2-fold increase in caspase-8 activities, respectively (Supplementary Figure S2A). Dominant negative FADD (DN-FADD, aa ∆80-293) transfection, in a dose-dependent manner, nearly totally blocked the increased caspase-8 activation induced by pCMV-p14-tBID (Supplementary Figure S2B). The mechanism of synergistic cell killing by p14-tBID involved both an increased extrinsic pathway (Fas L, caspase-8) and intrinsic apoptotic pathway components (increased Bax, Bak, PARP cleavage and decreased Bcl-2). This synergistic pro-apoptotic effect induced upon the expression of bicistronic p14/tBID is a novel finding.

To further confirm that the mechanism of cell death from bicistronic expression of p14 and tBID was through both the intrinsic and extrinsic apoptotic pathways, we tested inhibitors for Caspase-8, Caspase-9, and Caspase-3, each at their IC50 (2 µM), in PANC1 cancer cells transfected with Ad-pCMV-p14-tBID (Supplementary Figure S2C). In Annexin V assays, inhibitors of caspase-8, -9 and -3 alone reduced apoptosis by the following after subtraction of controls: 71% to 5% (93% decrease), 71% to 10% (86% decrease), and 71% to 6% (92% decrease), respectively (Supplementary Figure S2C). The combination of caspase-8 and -9 inhibitors or all three caspase inhibitors together did not significantly inhibit apoptosis in comparison to the inhibition induced by caspase-8 alone (93% decrease). Caspase-8 and -9 inhibitors together reduced apoptosis from 71% to 5% (95% decrease); all three inhibitors of caspase-8, -9, and -3 reduced apoptosis from 71% to 4% (94% decrease). These results confirmed that Ad-pCMV-p14-tBID induced both intrinsic and extrinsic death pathways and that the great majority (94%) (but not all) of the apoptosis was mediated by the extrinsic and intrinsic pathways. Studies utilizing the major ROS inhibitors did not demonstrate any further decrease in apoptosis above 95%. This suggested that the reactive oxygen species (ROS) pathways are not important to the mechanism of apoptosis by p14-tBID.

### 3.5. Animal Studies

#### 3.5.1. Xenograft Models

**Figure 5:**
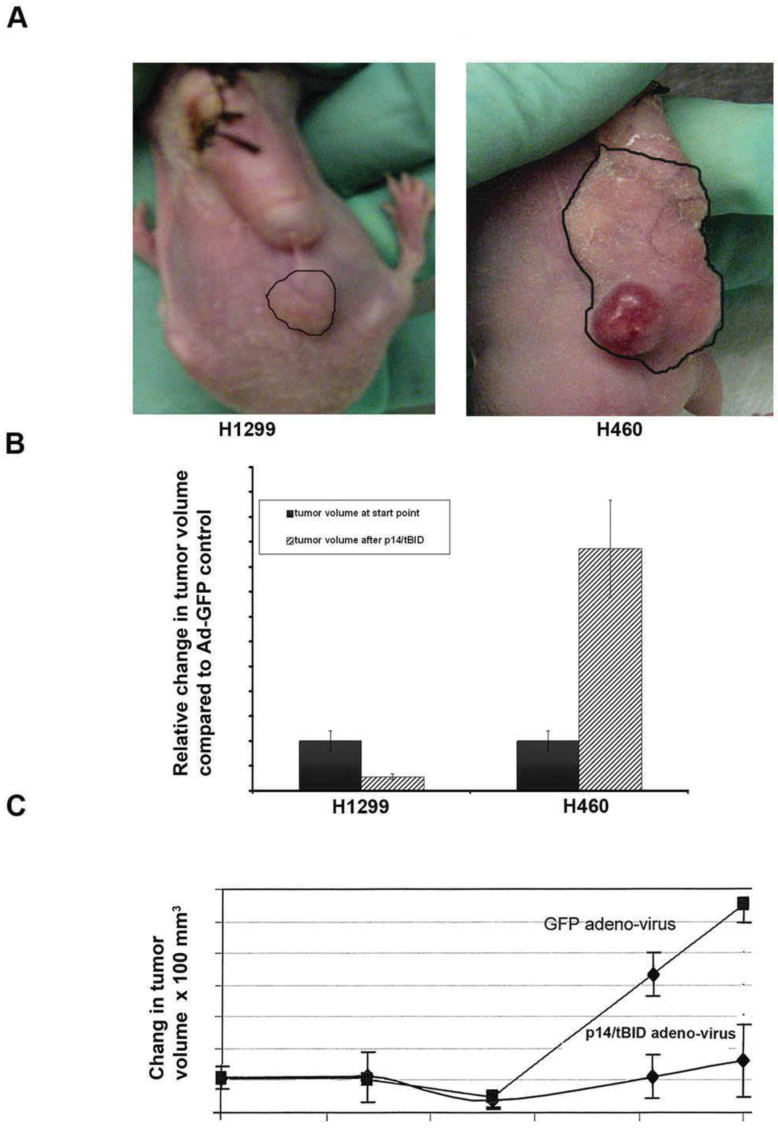
Human non-small cell lung cancer in nude mice models. (**A**) The tumor size of human lung cancer H1299 (null p53/mut N-Ras) and the H460 (wt p53/mut K-Ras) are demarcated by solid lines after injected Ad-p14ARF-MDR1n-p14- tBID for two weeks. (**B**) Relative change on tumor volume compared to the starting point after treated with anterior abdominal I.P. with Ad-p14ARF-MDR1n-p14-tBID for two weeks. H1299 group (n = 10) and H460 group (n = 11). In the H1299 group, the tumor average size has shrunk to 0.28 ± 0.17 fold compared to starting point whereas H460 lung group, tumor size has increased to 4.86 ± 0.198 fold compared to started point, as expected since H460 carries wt p53, whereas in H1299 groups receiving Ad-p14ARF-MDR1-p14-tBID tumor volumes significantly changed in both group compared to the starting point (Student’s *t*-test, *p* < 0.01). (**C**) H1229 xenograft average growth pattern. Control group animals treated with Ad-pCMV-GFP vs. the experimental group treated with Ad- p14ARF-MDR1-p14-tBID showing a significant decrease in tumor volume at day 25 (Student’s *t*-test, *p* < 0.001).

To study whether the construct p14^ARF^min-MDR1n-p14-tBID was active in vivo, lung cancer H1299 (mut N- Ras/null p53) and H460 (mut K-Ras/wt p53) cells were injected subcutaneously (1 × 10^6^ cells with matrigel) into the hind flank of female athymic (nude) mice. When the tumors were approximately 100 mm^3^, Alzet osmotic pumps were surgically implanted juxtaposed, but not in contact, with the tumors (Figure 5A). These pumps deliver 200 µL over a 14-day period of Ad-p14^ARF^min-MDR1n-p14-tBID (Ad-p14-tBID) or Ad-GFP or saline as controls. The solution contained 10^9^ virions/mL (2 × 10^8^ virions total in 200 µL). The volumes of the tumors after two weeks of treatment are shown in Figure 5A. H460 (mut Ras/wt p53) lung cancer tumor volumes were significantly larger than that of H1299 (mut N Ras/null p53) (Figure 5A, outlined areas). The local delivery of 200 µL of Ad-GFP did not alter tumor growth in either tumor type (data not shown). The H1299 group, which received local delivery of Ad-p14^ARF^min-MDR1n-p14-tBID, had final tumor volumes significantly smaller than the starting point (Figure 5B). This implied that the viral construct induced significant cytotoxicity and not just tumor stasis. In the control group, H460 lung cancer cell line (mut Ras/wt p53), showed no growth inhibition by the p14-tBID construct, and its growth pattern mimicked the controls of Ad-GFP or saline alone (not shown). This implied that the wt p53 status of the H460 tumor inhibited the p14-tBID construct (Figure 5B). The S.E.M bars are presented and do not overlap. To determine if the anti-tumor effect of the active construct could be demonstrated from a distal point of delivery and in larger tumors, we performed similar experiments with the H1229 cell line and constructs as previously described. However, this time IP administration was utilized in mice with SC tumor volumes starting at 200 µL of Ad-p14-tBID (experimental group) and Ad-GFP (control group) was delivered via IP every four days over 24 days (total of seven injections at a titer of 10^9^ virions/mL; Figure 5C). At day 25, the study was terminated due to progressive tumor growth in the control group. At end point, the control group tumor volume presented a 4.33-fold difference in tumor volume compared to the experimental group (1300 mm^3^ and 300 mm^3^ respectively). There was no significant difference between control and experimental groups using the H460 lung cancer cell line (mut Ras/wt p53). These results demonstrated the anti-tumor cytotoxicity and specificity of the construct in mice xenografted with a mut Ras/null p53 cell line regardless of the delivery mechanism.

#### 3.5.2. Pancreatic Cancer in Genetically Engineered Mouse Model (GEMM): Gene Therapy of GEMM (LSL-Kras^G12D/+^; LSL-Trp53R^172H/+^; Pdx-1-Cre (KPC)) mice with Ad-p14^ARF^min-MDR1n-p14-tBID

To further investigate the in vivo anti-tumor effects of the construct in a more stringent mouse model, we used the established KPC model with *K-Ras^G12D/+^* and *p53R^172H/+^* mutations (12,13). In this KPC genetic mouse model of pancreatic cancer, the mutant *K-Ras* and *p53* alleles are expressed in pancreatic cancer cells.

**Figure 6:**
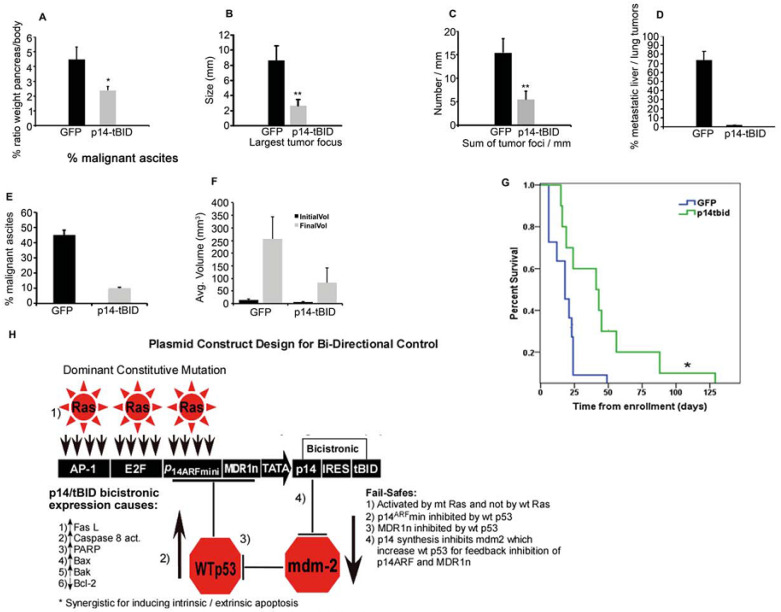
The p14-tBID treatment in the GEMM KPC model. (**A**) Percentage of pancreas-to-body weight ratios at sacrifice in mice treated from 4 months to 6 months of age (three times/week) with p14-tBID or control groups (mean ± SEM). (**B**) Average size in mm of the largest tumor focus in each of the KPC mice treated with p14-tBID or the control group (mean ± SEM). (**C**) Average number of tumor foci within a 1 mm field detected in the KPC mice treated with p14-tBID and the control group (mean ± SEM). (**D**) Metastatic tumor in the liver/lung expressed as percentage of p14-tBID-treated and control KPC mice (mean ± SEM). (**E**) Incidence of malignant ascites expressed as percentage of mice in the p14-tBID-treated group vs. controls group (mean ± SEM). (**F**) Average initial and final tumor volumes (measured by 3D ultrasound, in mm^3^) in p14-tBID-treated and control mice (mean ± SEM). (**G**) Kaplan–Meier survival curve for Ad-GFP-treated (*n* = 11, blue line) and p14-tBID-treated (*n* = 10, green line) KPC cohorts. A significant 2.3-fold increase in survival was observed in p14-tBID-treated (41 days-5.9 weeks) mice relative to the control cohort (18 days-2.6 weeks). (* *p* = 0.023, Log-rank test, HR = 0.314). (**H**) Plasmid construct, bi-directional control by fail-safes and anti-tumor effects through the intrinsic/extrinsic pathways of apoptosis. (* *p* < 0.05; ** *p* < 0.01; Student’s *t*-test).

Moreover, *p53* wild-type allele is usually lost, especially in the metastases. Mice (4 months of age) were IP injected three times a week for two months with 200 µL with the 10^9^/mL containing Ad-GFP (control) or Ad-p14^ARF^min-MDR1n-p14-tBID (p14-tBID) over 24 injections (2 × 10^8^ virions/injection). At this age (6 mo), virtually ≥90% of mice presented with gross pancreatic cancer, with ≥70% showing metastatic disease. After two months of treatment, animals were examined for the presence of tumors (Figure 6). Grossly, the entire pancreases of the control group contained many more tumor foci and larger solid tumor masses. This is reflected by the higher ratio of pancreas to body weight of the control group (*n* = 12; 4.86 ± 0.95 g) in comparison to the p14-tBID group (*n* = 12; 2.73 ± 0.14 g; Student’s *t*-test *p* = 0.04) (Figure 6A). By determining the sizes of the tumor foci of each tumor mass microscopically, we found that treatment with p14-tBID significantly reduced the size of the largest tumor focus in the p14-tBID treated mice vs. the control group, respectively (3.00 ± 0.85 mm and 9.61 ± 1.88 mm, respectively) (Student’s *t*-test, *p* = 0.003; Figure 6B). The sum of the entire tumor foci detected within a span of 1 mm of each pancreatic tumor sample was also decreased (5.47 ± 1.76 mm for p14-tBID vs. 15.38 ± 2.95 mm for the control group; Student *t*-test, *p* = 0.005; Figure 6C). These findings suggested that the p14-tBID treatment reduced the total tumor mass by reducing the sizes and numbers of tumor foci developed in mice with mut K-Ras and mut p53 by approximately three-fold. As for metastases, none of the p14-tBID-treated mice (0/10) developed liver or lung metastases, while 72.7% (8/11) of the controls had metastatic liver or lung tumor nodules (Figure 6D). Interestingly, malignant ascites (by microscopy) was more frequently found in the control group (45%; 5/11) than in the experimental group (10%; 1/10). This is a 78% decrease in the incidence of malignant ascites (Figure 6E).

These findings suggest that the p14-tBID treatment reduced the total tumor mass by reducing the sizes and numbers of tumor loci developed in each sample. The pronounced effect of the gene therapy in the liver/lung/peritoneal metastases may be related the loss of the wt alleles in the p53 and K-Ras genes such that the construct is fully activated.

Lastly, a survival study was performed in the KPC mice by methods previously described [14]. We monitored the tumor volume once a week by means of three-dimensional ultrasonography to determine the enrollment time. Mice were enrolled in the study when tumors grew to ≥2–5 mm (median 3 mm). Our results show that treatment with Ad-p14-tBid effectively reduced tumor volume at endpoint when compared with the control group treated with Ad-GDP (Figure 6F). Moreover, treatment with Ad-p14-tBID alone significantly extended the median survival of the KPC mice from 18 days to 41 days (Log-rank test, *p =* 0.023; Figure 6G). This translates to a 2.3-fold increase in median survival in mice treated with gene therapy as compared to control Ad-GFP with a hazard ratio (HR) of 0.314 ± 0.765 95% confidence interval (CI).

In both xenograft and GEMM models, treatment with Ad-p14-tBID did not produce discernible toxic side effects such as weight loss, hair texture, or morphologic changes in mice. Thus, the Ad-p14^ARF^-MDRln-p14-tBID clearly demonstrated significant antitumor efficacy in two xenograft models with proximal and distal sites of injection and in the KPC transgenic GEMM. Importantly, the active construct reduced tumor size and foci, completely abrogated metastases to the liver and lung, and significantly decreased the development of malignant ascites, increasing the overall median survival. Figure 6H shows a diagram of the construct with its bi-directional controls (inhibited by wt p53 and stimulated by mut Ras), its effect on the intrinsic/extrinsic pathways of apoptosis, and its four fail-safes to prevent activation in cells with either wt Ras or wt p53.

## 4. Discussion

Through a series of stepwise additions, we constructed a bidirectionally controlled promoter that is activated by mut Ras 11-fold higher than wt Ras (220% vs. 20% respectively). Mut Ras stimulated the AP-1e and E2Fe response enhancers and the p14^ARF^ minimal promoter, whereas wt p53 potently repressed the promoter through the negative regulatory elements of MDR1n and upon the p14^ARF^ minimal promoter. This bidirectionally modulated and modified p14^ARF^min promoter were linked to the bicistronic for p14 and tBID genes so that the entire construct can be small enough to be delivered by the adenovirus family and other small viral and non-viral vectors (total construct = 2.3 kb). Through the Cis-element Cluster finder promoter analysis program (Boston University), we added a TATA box to improve initiation of transcription. This promoter is preferentially activated by mut Ras and not by wt Ras.

The choice of the bicistronic genes (p14-IRES-tBID) was derived from experiments, which demonstrated maximal synergistic induction of apoptosis and activation of both extrinsic and intrinsic pathways of apoptosis. The rationale for using the *P14* gene was as follows: if the construct was inadvertently activated in normal cells with highly active Ras but low levels of wt p53, the increased p14 protein would directly inhibit HDM-2 mediated ubiquination and proteosomic degradation of wt p53. This leads to increased wt p53 levels, which would feedback and inhibit the p14^ARF^min promoter and the MDR1n element leading to cessation of construct activity. As demonstrated in the 24 cell lines with mut or wt Ras and p53, only those with both mut Ras and mut p53 were induced into apoptosis irrespective of the mut Ras status (K-, H-, and N-Ras). Cell lines with mut p53/mut K, N, H-Ras were induced into similar levels of apoptosis by the construct, which implied the subgroup of Ras mutation is not important. However, non-malignant cells, normal bone marrow stem cell progenitors (CFU- GEMM), and malignant lines with wt Ras/wt p53, mut Ras/wt p53, or wt Ras/mut p53 were not affected by the construct. The lack of cytotoxicity to malignant cells with wt Ras or wt p53 limits the clinical application of this construct as a gene therapy to cancers with concomitant *RAS* and *P53* mutations (e.g., pancreatic), but this was purposely arranged to increase the threshold for activation and safety margin of the construct in normal or non-malignant cells. (See Supplementary Table S1A,B for cell lines treated with the different constructs and mutational status or non-malignant cell lines respectively).

Overall, our data show that the CMV promoter linked to the p14-tBID construct induced substantial degrees of apoptosis irrespective of the *RAS* or *P53* status and malignant vs. non-malignant phenotypes, as expected. The modified p14^ARF^min promoter linked to the p14-tBID construct on the other hand induced approximately equal levels of apoptosis with or without the MDRn1 inhibitory element as the pCMV-driven construct in cell lines carrying mut Ras/mut p53. However, the levels of apoptosis were minimal in malignant cell lines, with either wt Ras or wt p53. Moreover, these levels were further suppressed to basal levels (two to five percent) by adding the MDR1n element. In non-malignant lines and in normal marrow stem cells, the levels of apoptotic induction by the construct with the MDR1n element were essentially equivalent to basal control levels. Thus, our strategy allows for a high degree of specificity and nearly absent levels of toxicity to cells with wt p53 and wt Ras and implies that the construct with MDR1n is inactive in malignant cells with wt p53 and/or wt Ras and is active only if there is a mut Ras/mut p53 genotype. Additionally, our data suggested that the construct was not active in normal cells with active wt Ras, such as marrow stem cells and non-malignant colon, breast, and skin cell lines undergoing active proliferation. Even in the MDA-MB 231 breast cancer cell line with heterozygous Ras (wt/mut K-Ras) and mut p53, the construct was not activated (Supplementary Figure S1B and Supplementary Table S1).

The high degree of specificity of the construct was precisely designed to prevent activation in non-malignant cells to avoid normal cell toxicity at the expense of losing an anti-tumor effect in malignant cells with either wt Ras and/or wt p53 genotypes. There are no known non-malignant cells that contain both mut Ras and mut p53, only pre-malignant and malignant cell types.

This construct was designed with four fail-safe mechanisms which prevent activation in normal or non-malignant cells without mut Ras and mut p53: (1) basal levels of wt p53 inhibit the modified p14^ARF^min promoter; (2) basal levels of wt p53 potently inhibit the MDR1n element; (3) induction of the *P14* gene, in cells with low basal levels of wt p53 and mut K-Ras, increases levels of wt p53 which feedback to further inhibit the modified p14^ARF^min promoter and the MDR1n element; and (4) constitutively activated mut K, N, or H-Ras activates the modified p14^ARF^min and the AP-1 and E2F enhancers except for wt Ras, such as in proliferating non-malignant or marrow stem cells do not activate the promoter to a significant level.

The other novel aspect of this construct is its genetic components. We found that the human *P14^ARF^* gene and the activated, truncated segment of BID (tBID), when concomitantly expressed, synergistically induced intrinsic and extrinsic apoptosis in many different types of cancer cells. This synergistic cell kill mediated by the bicistronic expression of p14 and tBID is a novel finding not previously reported. Components of the extrinsic pathway (increased Fas-L and caspase-8) and the intrinsic pathway (increased Bax, Bak, PARP cleavage, and decreased Bcl-2) are mechanistically involved, but how p14 and tBID mediate this synergy is unknown and warrants further investigation (Figure 6H) [18,19,20,21].

In comparison to previously published p53-containing gene therapies, this construct has selective advantages: (1) some p53-based gene therapies have a constitutive CMV promoter which does not differentiate between normal and cancer cells and can not be bi-directionally controlled; (2) p21^Waf/Cip-1^ induction by wt p53-based gene therapies can induce drug resistance to cell cycle active chemotherapies via cell cycle arrest through p21, which was not induced by our construct [22]; (3) bidirectional control of the p14^ARF^min-modified promoter is activated only in cells with mut Ras and non-functional p53; and (4) the concomitant expression of p14 and tBID act synergistically and mechanistically through the extrinsic and intrinsic pathways of apoptosis only in cells with both mut Ras and mut or null p53. Together, these characteristics greatly increase specificity and safety.

In both xenograft and GEMM models, treatment with Ad-p14-tBID did not produce discernible toxic side effects such as weight loss or hair texture or morphologic changes in the mice. Thus, the Ad-p14^ARF^min-MDR1n-p14-tBID clearly demonstrated significant antitumor efficacy in two xenograft models with proximal and distal sites of injection and in the KPC transgenic GEMM. Importantly, the active construct not only reduced tumor size and foci but completely abrogated metastases to the liver and lung and significantly decreased the incidence of malignant ascites, increasing the overall median survival.

More importantly, the ability of this active construct was demonstrated in two xenograft models with proximal and distant delivery and in the KPC pancreatic cancer mouse model, where there was a 2.3-fold increased median survival: 18 days (2.5 weeks) for control and 41 days (5.9 weeks) for the p14-tBID group.

In summary, the incidence of the concomitant mut Ras/mut p53 genotype is high in pancreatic cancers (70–80%) as well as in selected cancers such as bladder (70%), NSCLC, and colon (all 30–35%) [23,24]. The subtype of Ras mutation (K, N, or H) is not a determinant for activation of the promoter. Our in vitro data demonstrated that the endogenous mut N-Ras (lung) and H-Ras (bladder) also stimulate the promoter as well as mut K-Ras in pancreatic cancer (Figure 3 and Supplementary Table S1A).

## 5. Future Perspectives

The translation of this construct could potentially be extended to other malignances that have a substantial percentage of mut Ras/mut p53.

## Data Availability

Available upon request by primary corresponding author.

## Supplementary Materials

The following supporting information can be downloaded at: https://www.mdpi.com/article/10.3390/biomedicines11020258/s1, Table S1: Characteristics of Adenovirus Vector Treated Cell Lines. Figure S1: A. Malignant cell lines with mut Ras/wt p53. HCT116 (colon) and H460 (lung) have mut Ras/wt p53. Cells harvested 48 h. after infection (20 MOI) were analyzed for apoptosis by PI staining. The pCMV-p14-tBID construct induced apoptosis in the range of 38% to 56%; the p14ARF-p14-tBID induced apoptosis in the range of 3% to 20%; the p14ARF-MDR1n-p14-tBID induced apoptosis from 2% to 10%. B. Malignant cell lines with wt Ras/mut p53. BxPC-3 (pancreatic, wt Ras/mut p53), MB231 (breast, heterozygosity (wt/mut) Ras)/mut p53) and A431 (squamous skin, wt Ras/mut p53) cells were harvested 48 h after infection and were subjected to flow cytometric detection of apoptosis by PI staining. The pCMV-p14- tBID construct induced apoptosis in the range of 49% to 85%, while modified p14ARFmin-MDR1n-p14-tBID construct induced apoptosis of 2% to 4% (MOI = 50). C. Malignant cell lines with wt Ras/wt p53. MSTO-211H (mesothelioma), MCF7 (breast) and HEAND (liver angiosarcoma) have wt Ras/wt p53. Cells were harvested 48 h after infection and subjected to flow cytometric detection of apoptosis by PI staining. pCMV-p14-tBID induced apoptosis in the range of 41% to 55%; p14ARF-MDR1n-p14-tBID induced apoptosis from 3–5% (MOI = 50). Figure S2: The pCMV-p14-tBID construct induced intrinsic and extrinsic apoptosis. A. Extrinsic pathway caspase-8 activity. H1229 lung cells (mut N-Ras/null p53) were infected with 5 MOI of each virus containing respective vector, and tested for caspase-8 activity using a colorimetric assay Kit (MBL, Woburn, MA). The pCMV and p14ARF driven constructs containing p14 and tBID ± MDR1n all induced approximately the same fold increase in caspase 8 activity (6.2 to 6.9 fold). Also, the concomitant expression of p14 and tBID induced a synergistic increase in caspase-8 activity (6.9 fold) as compared to pCMV-p14 (1.1 fold) and pCMV-tBID alone (4.2 fold). B. Induction of caspase-8 activity by pCMV-p14-tBID is inhibited by dominant negative FADD (DN-FADD). H1299 lung cells (mut N-Ras/null p53) were infected with 5 MOI of each virus containing empty vector and DN-FADD (aa 80-293) vector for 8 h. and secondarily infected with 5 MOI of pCMV-p14-tBID for another 16 h, then tested for caspase-8 activity. In a dose dependent manner, DN-FADD blocked 75% of maximal caspase-8 activity when stimulated by the pCMV-p14-tBID construct. C. Inhibition of Caspases-3, 8, and 9 blocks apoptosis induced by pCMV-p14-tBID. H1229 lung cancer cells (mut N-Ras/null p53) were infected with 5 MOI adenovirus for 6 h; then either Caspase-3 inhibitor (Z-DEVD-FMK), or Caspase-8 inhibitor (Z-IETD-FMK) or Caspase-9 inhibitor (Z-LEHD-FMK) (MBL, Woburn, MA, USA) were added at their IC50 (2 M) and in combinations for another 18 h. Cells were harvested and stained with annexin V (A13201, Invitrogen, Corp., Carlsbad, CA, USA) and analyzed by FACS. Caspase-8 inhibitor alone suppressed pCMV-p14-tBID induced apoptosis from 75% to 9%; caspase-9 inhibitor suppressed apoptosis from 75% to 14% and caspase 3 inhibitor suppressed apoptosis from 75% to 11%. Combinations of all 3 caspase inhibitors suppressed apoptosis from of 75% to 8%.
