# Supplementary material for: Gene Therapy with p14/tBID Induces Selective and Synergistic Apoptosis in Mutant Ras and Mutant p53 Cancer Cells In Vitro and In Vivo
Source: Biomedicines. 2023 Jan 18;11(2):258. doi: 10.3390/biomedicines11020258 (PMC9953161; doi:10.3390/biomedicines11020258)
Supplement: Supplementary file 1 [file biomedicines-11-00258-s001.zip › biomedicines-1709282-supplementary.pdf]

biomedicines

Table S1. Characteristics of Adenovirus Vector Treated Cell Lines.

Table S1A. Characteristics of Human Malignant Cell Lines.

Cell Line	Derived Tissue	Ras Status	p53 Status	pCMV-p14-tBID Toxicity	$p14^{ARF}$-p14-tBID Toxicity	$p14^{ARF}$MDR1n-p14-tBID Toxicity
BxPC-3	pancreatic	wild type	mutant	49%	3%	3%
PANC-1	pancreatic	mutant K	mutant	54%	52%	53%
AsPC-1	pancreatic	mutant K	mutant	42%	31%	31%
H1299	Lung	mutant N	null	62%	43%	43%
H1299 / ts p53-#143	Lung	mutant N	mutant at 37°C	69%	43%	46%
			wild type at 32°C	50%	13%	8%
H460	Lung	mutant K	wild type	56%	3%	2%
T24	bladder	mutant H	mutant	43%	30%	32%
ISO-Has	angiosarcoma	mutant K	mutant	35%	30%	32%
HEAND	angiosarcoma	wild type	wild type	59%	6%	3%
MSTO-211H	mesothelioma	wild type	wild type	41%	3%	3%
HCT 116	colon	mutant K	wild type	38%	20%	10%
MCF-7	breast	wild type	wild type	50%	10%	5%
MB231	breast	mutant k / wt	mutant	85%	4%	2%
A431	Skin	wild type	mutant	59%	4%	4%
PC-3 / ts p53-#143	prostate	mutant K	mutant at 37°C	51%	36%	37%
			wild type at 32°C	54%	10%	6%

All lines were grown and treated in exponential growth with Adenovirus 5 vector.

Table S1B. Characteristics of Human Non-Malignant Cell Lines.

Cell Line	Derived Tissue	Ras Status	p53 Status	pCMV-p14-tBID Toxicity	$p14^{ARF}$-p14-tBID Toxicity	$p14^{ARF}$MDR1n-p14-tBID Toxicity
MCF-10F	breast	wild type	wild type	50%	12%	3%
MCF10-2A	breast	wild type	wild type	37%	10%	2%
CCD-33Co	colon	wild type	wild type	31%	7%	4%
CCD-27sk	skin fibroblast	wild type	wild type	36%	9%	3%
BFU-E	marrow stem	wild type	wild type	12%	3%	3%
CFU-GMM	marrow stem	wild type	wild type	42%	6%	6%

All lines were grown and treated in exponential growth with Adenovirus 5 vector. * Ad 5 vector alone had 5% toxicity to BFU-E and CFU-GMM colonies.

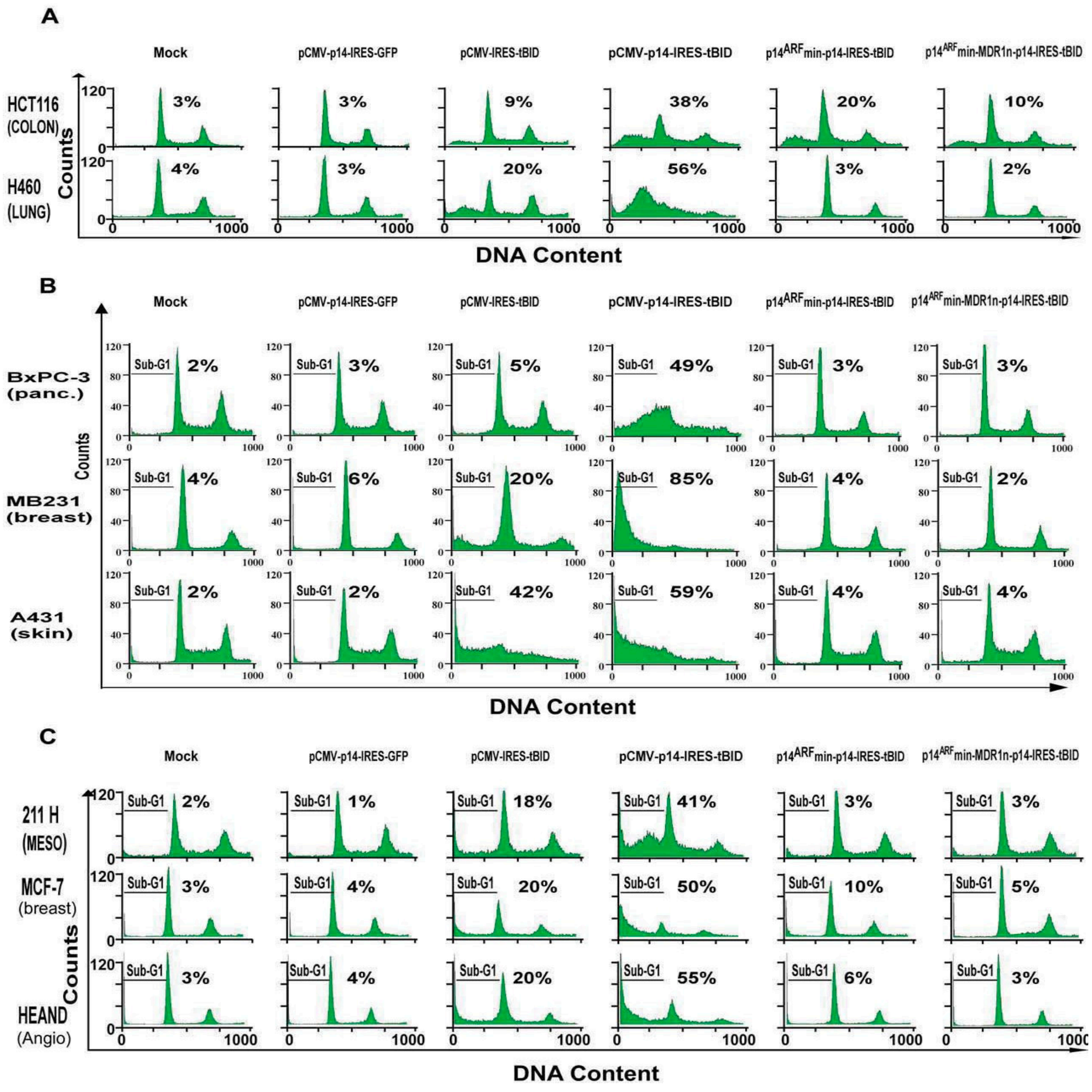

Figure S1: (**A)** Malignant cell lines with mut Ras/wt p53. HCT116 (colon) and H460 (lung) have mut Ras/wt p53. Cells harvested 48 h. after infection (20 MOI) were analyzed for apoptosis by PI staining. The pCMV-p14-tBID construct induced apoptosis in the range of 38% to 56%; the p14ARF-p14-tBID induced apoptosis in the range of 3% to 20%; the p14ARF-MDR1n-p14-tBID induced apoptosis from 2% to 10%. (**B)** Malignant cell lines with wt Ras / mut p53. BxPC-3 (pancreatic, wt Ras/mut p53), MB231 (breast, heterozygosity (wt/mut) Ras)/mut p53) and A431 (squamous skin, wt Ras/mut p53) cells were harvested 48 h after infection and were subjected to flow cytometric detection of apoptosis by PI staining. The pCMV-p14- tBID construct induced apoptosis in the range of 49% to 85%, while modified p14ARFmin-MDR1n-p14-tBID construct induced apoptosis of 2% to 4% (MOI = 50). C. Malignant cell lines with wt Ras/wt p53. MSTO-211H (mesothelioma), MCF7 (breast) and HEAND (liver angiosarcoma) have wt Ras/wt p53. Cells were harvested 48 h after infection and subjected to flow cytometric detection of apoptosis by PI staining. pCMV-p14-tBID induced apoptosis in the range of 41% to 55%; p14ARF-MDR1n-p14-tBID induced apoptosis from 3%-5% (MOI = 50).

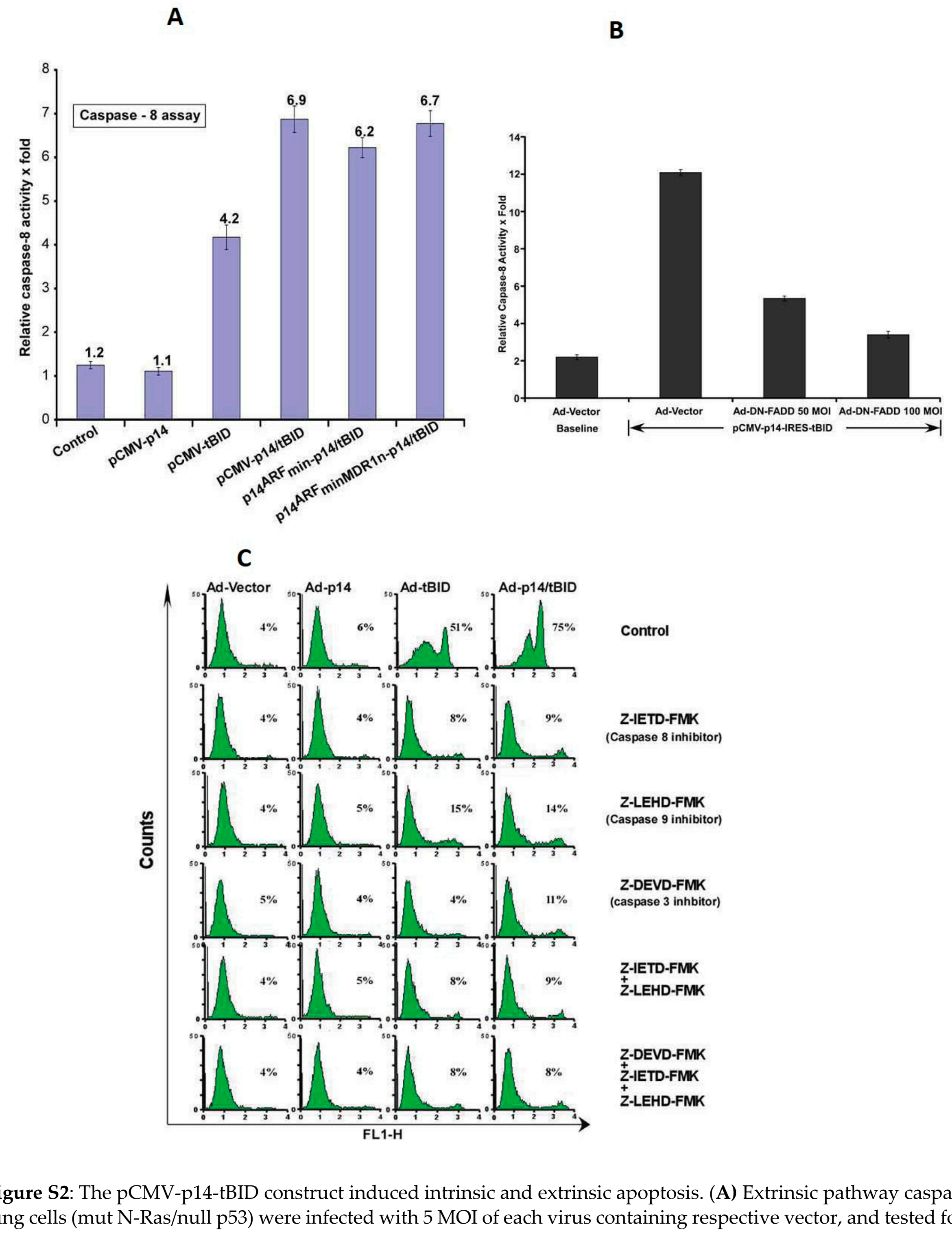

Figure S2: The pCMV-p14-tBID construct induced intrinsic and extrinsic apoptosis. (**A)** Extrinsic pathway caspase-8 activity. H1229 lung cells (mut N-Ras/null p53) were infected with 5 MOI of each virus containing respective vector, and tested for caspase-8 activity using a colorimetric assay Kit (MBL, Woburn, MA). The pCMV and p14ARF driven constructs containing p14 and tBID ± MDR1n all induced approximately the same fold increase in caspase 8 activity (6.2 to 6.9 fold). Also, the concomitant expression of p14 and tBID induced a synergistic increase in caspase-8 activity (6.9 fold) as compared to pCMV-p14 (1.1 fold) and pCMV-tBID alone (4.2 fold). (**B)** Induction of caspase-8 activity by pCMV-p14-tBID is inhibited by dominant negative FADD (DN-FADD). H1299 lung cells (mut N-Ras/null p53) were infected with 5 MOI of each virus containing empty vector and DN-FADD (aa 80-293) vector for 8 h. and secondarily infected with 5 MOI of pCMV-p14-tBID for another 16 h, then tested for caspase-8 activity. In a dose dependent manner, DN-FADD blocked 75% of maximal caspase-8 activity when stimulated by the pCMV-p14-tBID construct. (**C)** Inhibition of Caspases-3, 8, and 9 blocks apoptosis induced by pCMV-p14-tBID. H1229 lung cancer cells (mut N-Ras/null p53) were infected with 5 MOI adenovirus for 6 h; then either Caspase-3 inhibitor (Z-DEVD-FMK), or Caspase-8 inhibitor (Z-IETD-FMK) or Caspase-9 inhibitor (Z-LEHD-FMK) (MBL, Woburn, MA) were added at their IC50 (2 M) and in combinations for another 18 h. Cells were harvested and

stained with annexin V (A13201, Invitrogen, Corp., Carlsbad, CA) and analyzed by FACS. Caspase-8 inhibitor alone suppressed pCMV-p14-tBID induced apoptosis from 75% to 9%; caspase-9 inhibitor suppressed apoptosis from 75% to 14% and caspase 3 inhibitor suppressed apoptosis from 75% to 11%. Combinations of all 3 caspase inhibitors suppressed apoptosis from of 75% to 8%.
